# Chromatin remodeler Znhit1 controls bone morphogenetic protein signaling in embryonic lung tissue branching

**DOI:** 10.1016/j.jbc.2022.102490

**Published:** 2022-09-14

**Authors:** Wei Wei, Xiaofang Tang, Ning Jiang, Chao Ni, Hua He, Shenfei Sun, Meng Yu, Chuyue Yu, Mengdi Qiu, Dong Yan, Zhaocai Zhou, Yuanlin Song, Hanmin Liu, Bing Zhao, Xinhua Lin

**Affiliations:** 1 State Key Laboratory of Genetic Engineering, School of Life Sciences, Greater Bay Area Institute of Precision Medicine (Guangzhou), Zhongshan Hospital, Fudan University, Shanghai, China; 2 The Joint Laboratory for Lung Development and Related Diseases of West China Second University Hospital, Sichuan University and School of Life Sciences of Fudan University, Chengdu, China; 3 Department of Pulmonary Medicine, Zhongshan Hospital, Fudan University, Shanghai, China; 4 Shanghai Key Laboratory of Lung Inflammation and Injury, Shanghai, China

**Keywords:** chromatin remodeling, lung development, branching morphogenesis, Znhit1, Bmp4, BMP, bone morphogenetic protein, CCSP, club cell secretory protein, ChIP, chromatin immunoprecipitation, COPD, chronic obstructive pulmonary disease, DMEM, Dulbecco's modified Eagle's medium, E9, embryonic day 9, FGF, fibroblast growth factor, PH3, phosphorylated histone H3, pSMAD1/5/9, phosphor-SMAD1/5/9, qRT–PCR, quantitative RT–PCR, Znhit1, zinc finger HIT-type containing 1

## Abstract

Branching morphogenesis is a key process essential for lung and other organ development in which cellular and tissue architecture branch out to maximize surface area. While this process is known to be regulated by differential gene expression of ligands and receptors, how chromatin remodeling regulates this process remains unclear. Znhit1 (zinc finger HIT-type containing 1), acting as a chromatin remodeler, has previously been shown to control the deposition of the histone variant H2A.Z. Here, we demonstrate that Znhit1 also plays an important role in regulating lung branching. Using *Znhit1* conditional KO mice, we show that *Znhit1* deficiency in the embryonic lung epithelium leads to failure of branching morphogenesis and neonatal lethality, which is accompanied by reduced cell proliferation and increased cell apoptosis of the epithelium. The results from the transcriptome and the chromatin immunoprecipitation assay reveal that this is partially regulated by the derepression of *Bmp4*, encoding bone morphogenetic protein (BMP) 4, which is a direct target of H2A.Z. Furthermore, we show that inhibition of BMP signaling by the protein inhibitor Noggin rescues the lung branching defects of *Znhit1* mutants *ex vivo*. Taken together, our study identifies the critical role of Znhit1/H2A.Z in embryonic lung morphogenesis *via* the regulation of BMP signaling.

The lung is one of the essential organs that terrestrial vertebrates have evolved to adapt to life on land. The complex architecture of the lung enables the vital function of gas exchange, and lung development entails a series of remarkably elaborate branching events from the primary lung buds (1). In mice, the lung is derived from the ventral anterior foregut endoderm and appears at embryonic day 9 (E9) (2). The primary lung buds are formed at the embryonic stage E9.5–E12.5. During the subsequent pseudoglandular stage (E12.5–E16.5), the lung forms the primary airway pattern. SOX9-expressing proximal epithelial progenitors give rise to bronchioles and gas exchange units, and SOX2-expressing proximal regions gradually develop into the conductive airways in the mature lung. The functional adult lung is progressively formed after canalicular, saccular, and final alveolar stages.

The pattern formation of the lung is controlled by a series of important signaling molecules, including bone morphogenetic protein 4 (BMP4), SHH, fibroblast growth factor 10 (FGF10), and wingless (Wnt). Interactions between FGFs and BMPs are crucial to the formation of secondary lung buds. BMP4-deficient mouse embryos die before E10, whereas the excessive activation of BMP4 antagonizes the FGF10-induced epithelial bud growth (3, 4, 5). These signaling molecules working together with their downstream target genes regulate lung morphological development. In addition, epigenetic regulations, including DNA methylation, histone modification, and chromatin remodeling, also play vital roles in the regulation of gene expression during lung development.

Chromatin remodeling is tightly coupled to lung branching. Loss of histone deacetylase 1/2 (5) or Sin3a (6), two components of the NuRD chromatin remodeling complex, impairs proximal airway epithelial differentiation partially through the activation of Bmp4 during mouse lung endoderm development. These findings suggest that chromatin remodeling is indispensable for proximal progenitor differentiation during lung development.

The replacement of canonical histone H2A with histone variant H2A.Z leads to chromatin remodeling and subsequent gene expression changes (7, 8, 9, 10, 11). As a key subunit of the SNF-2 related CBP activator protein complex, the zinc finger HIT-type containing 1 (Znhit1) mediates the exchange of histone variant H2A.Z for H2A. Our recent studies have demonstrated that Znhit1 incorporates H2A.Z for transcriptional regulation of genes in Lgr5-positive stem cell maintenance (12), hematopoietic homeostasis (13), and meiotic initiation (14). However, the function of Znhit1/H2A.Z in mammalian pulmonary development remains unknown.

In this study, we show that *Znhit1* deficiency in embryonic respiratory epithelial cells results in severe branching defects, accompanied by reduced proliferation and increased apoptosis of the entire lung epithelia. The deletion of *Znhit1* leads to increased expression of *Bmp4* in lung epithelium and overactivation of its downstream target SMAD; also the incorporation of H2A.Z in *Bmp4* promoter region is Znhit1 dependent. Inhibition of BMP4 signaling in 3D lung epithelial organoids with the BMP antagonist Noggin can partially rescue the branching defects as shown by enhanced bud formation. Taken together, our work demonstrates that the epigenetic factor Znhit1 acts as a repressor of *Bmp4* expression through H2A.Z in the embryonic epithelium during lung branching morphogenesis.

## Results

### Znhit1 deletion impairs embryonic lung epithelium development

**Figure 1:**
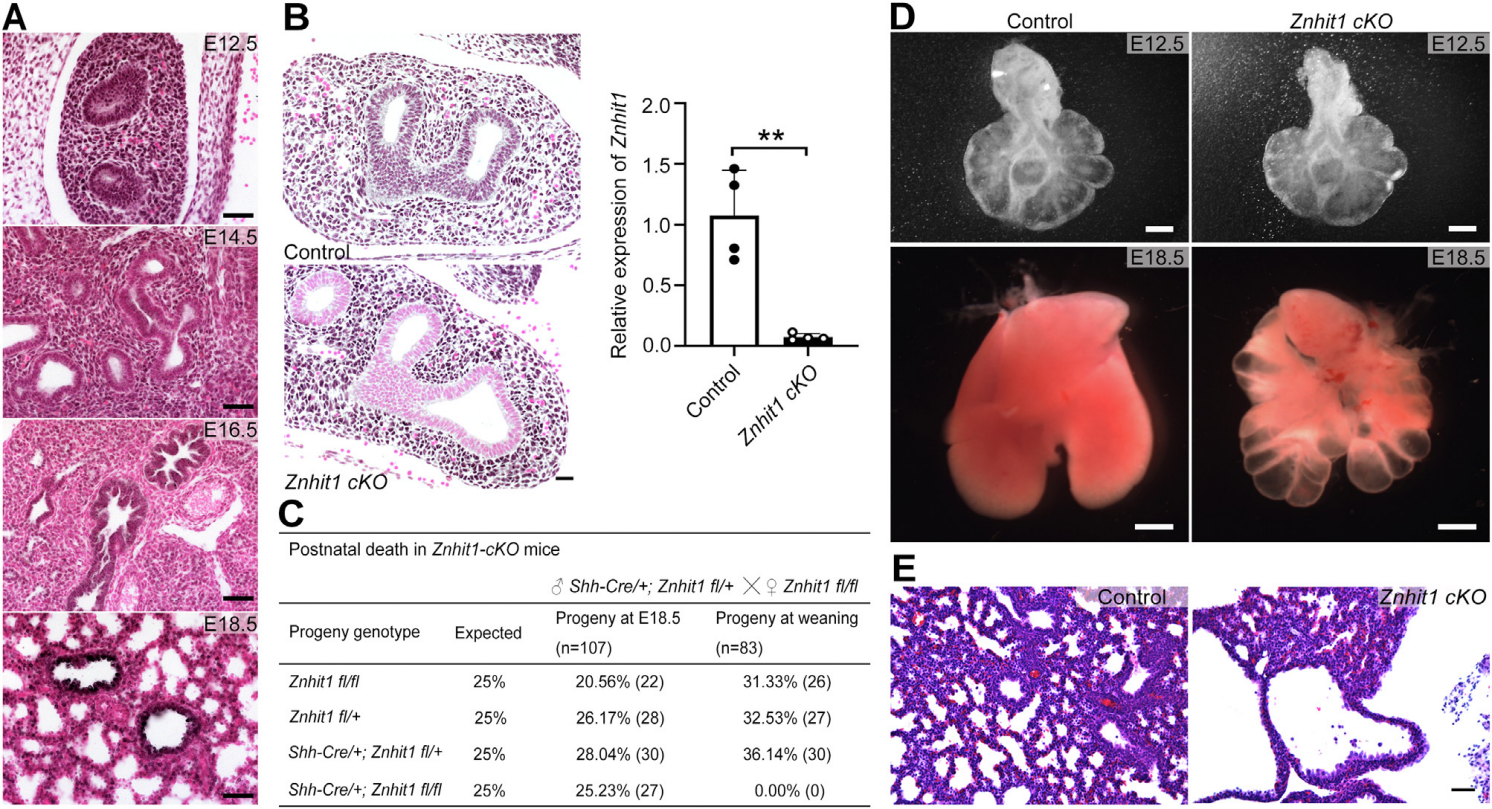
***Znhit1* deletion in embryonic lung epithelium impairs prenatal development.***A*, the expression pattern of Znhit1 is showing revealing dynamic changes of Znhit1 expression during lung prenatal development in mice (embryonic day; E12.5, E14.5, E16.5, and E18.5). The scale bars represent 200 μm. *B*, the high efficiency of *Znhit1* KO driven by Shh-Cre was proved by immunohistochemical staining at E12.5. Quantitative RT–PCR (qRT–PCR) of RNA extracted from E13.5 control and *Znhit1*-deficient EpCAM+ sorted lung epithelial cells confirmed a significant decrease of *Znhit1* expression in homozygous *Znhit1* conditional KO (*cKO*) mice. Means ± SEM; ∗∗*p* < 0.01 using unpaired two-tailed Student’s *t* test. n = 4/group. The scale bars represent 100 μm. *C*, statistic table of postnatal death in *Znhit1 cKO* group. *D*, lungs from control and *Znhit1 cKO* mice (E18.5) are shown demonstrating cystic defects after deletion of *Znhit1*. The scale bars represent 200 μm (E12.5) and 1000 μm (E18.5). *E*, hematoxylin/eosin-stained sections of control and *Znhit1* mutant lungs at E18.5. The scale bars represent 50 μm. Znhit1, zinc finger HIT-type containing 1.

To determine the functions of Znhit1 in lung development, we first examined the expression pattern of Znhit1 during fetal lung development by immunochemistry histological staining. As shown in Fig. 1*A*, Znhit1 was widely expressed throughout the lung from E12.5 to E18.5 with nuclear localization.

To assess the potential role(s) of Znhit1 in epithelium development, we used *Shh-Cre* (15) to delete *Znhit1* in the respiratory epithelium before birth. The efficiency of Cre-mediated epithelial deletion of *Znhit1* was examined through quantitative RT–PCR (qRT–PCR) and immunostaining. The expression of Znhit1 was absent in the mutant lung epithelium at E12.5 but remained normal in the rest of the lung (Fig. 1*B*). The survival of *Shh-Cre; Znhit1*^*fl/fl*^ (referred to as “*Znhit1 cKO*” [conditional KO] hereafter) mice during embryogenesis was as good as those of *Znhit1*^*fl/fl*^ and *Shh-Cre; Znhit1*^*fl/+*^ (referred to as “control” hereafter) mice, with no significant weight/length changes (Figs. 1*C*; S1). While *Znhit1*^*fl/fl*^ and control mice both survived normally without any observable malformations, *Znhit1*-mutant neonatal mice died of respiratory failure immediately after birth. Further analysis of fetal lung development revealed that *Znhit1 cKO* embryos did form primary lung buds at E12.5 but exhibited multiple distal cysts instead of well-formed lung tissue by E18.5 (Figs. 1, *D* and *E*; S2), indicating an important function of Znhit1 in epithelial pattern formation.

### Znhit1 is essential for embryonic lung distal epithelium branching

**Figure 2:**
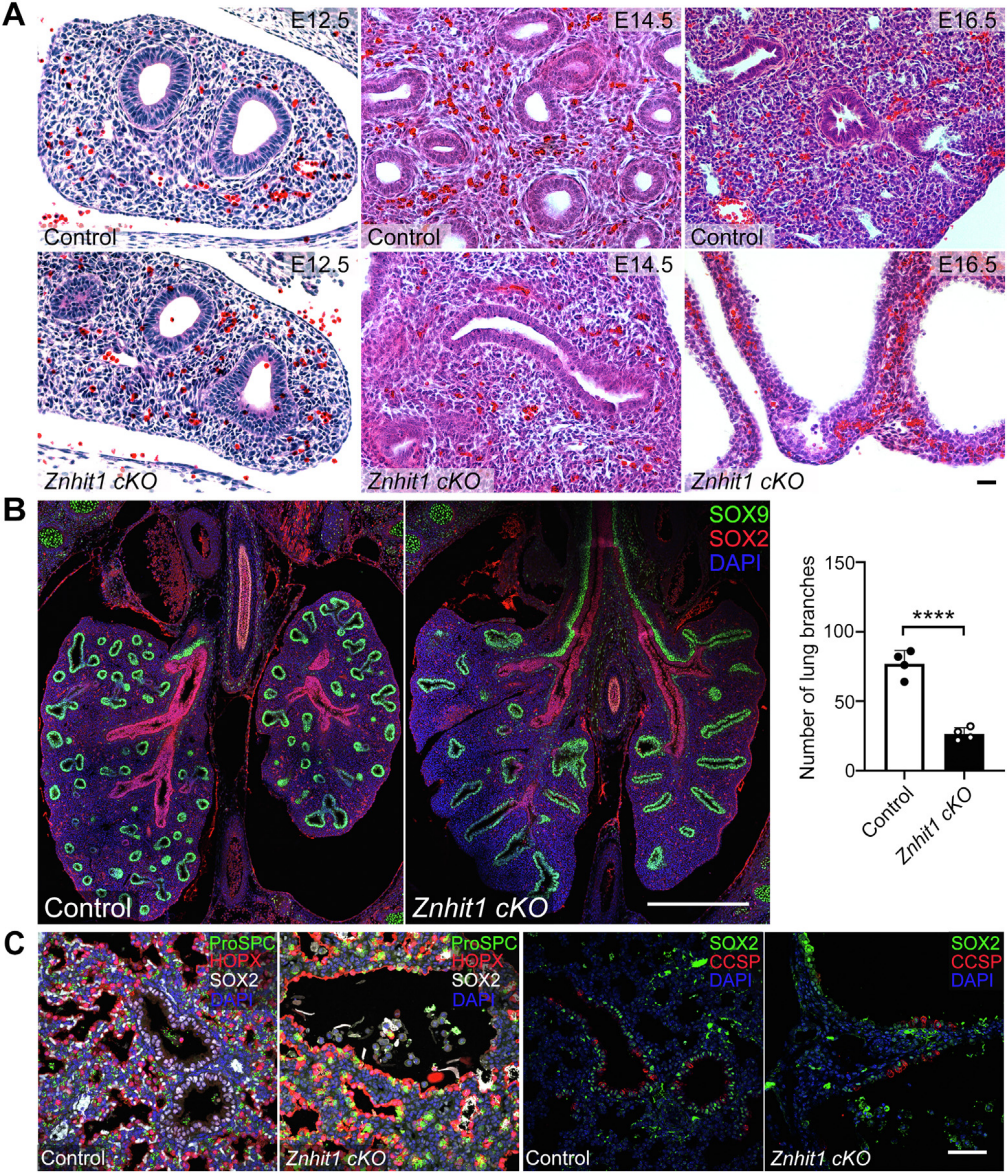
**Znhit1 is essential for embryonic lung distal epithelium branching.***A*, hematoxylin/eosin-stained sections reveal normal lung morphogenesis at embryonic day 12.5. (E12.5) but show branching defects at E14.5 and cystic lung structure at E16.5. The scale bars represent 100 μm. *B*, E14.5 control and *Znhit1* conditional KO (*cKO*) lung sections were stained with SOX2 and SOX9. SOX9-positive cells were showing an extended distal pattern and fewer branches in *Znhit1*-deficient epithelium. The scale bars represent 500 μm. Quantification of SOX9-positive branches at E14.5. Means ± SEM; ∗∗∗∗*p* < 0.0001 using unpaired two-tailed Student’s *t* test. n = 4/group. *C*, E18.5 control and mutant lung sections were stained with SOX2 (proximal lung epithelial progenitor marker), proSPC (AT2 marker), HOPX (AT1 marker), and CCSP (club cell marker). *Znhit1*-deficient lungs show differentiation of distal epithelial cells but less SOX2-positive progenitors the day before birth. The scale bars represent 50 μm. Znhit1, zinc finger HIT-type containing 1.

To determine whether Znhit1 regulates lung branching, we performed hematoxylin and eosin staining with lung sections of E12.5, E14.5, and E16.5. The observable branching defects at E14.5 developed into cystic morphology by E16.5 (Fig. 2*A*). Along the proximodistal axis during branching morphogenesis, the lung endoderm with SOX2 expression marks the proximal airway progenitors (16, 17), whereas SOX9 marks the distal progenitors (18, 19). In E14.5 sections, the SOX2+ region aggressively extended and the SOX9+ region was restricted in the distal epithelial tips accompanied by branching morphogenesis in control mice. But the SOX9+ area showed an extended distal pattern and fewer branches in *Znhit1*-deficient epithelium while the patterning of the SOX2+ region remained largely unchanged (Fig. 2*B*).

The abnormal branching patterns of *Znhit1* mutant mice suggest the possible functions of Znhit1 in regulating cell differentiation, proliferation, and cell death. Cell differentiation events during lung cell lineage specifications have been shown to regulate branching morphogenesis (20). To test whether the branching defects of *Znhit1* mutant in the epithelium are due to its role in cell differentiation, we examined markers for major cell types of lung epithelium at E18.5. Airway club cell differentiation indicated by club cell secretory protein (CCSP), as well as AT1 cells and AT2 cells labeled by HOPX and proSPC staining, was undisturbed (Fig. 2*C*). The SOX2+ cells were lost in *Znhit1*-deficient lungs at E18.5 but remained unchanged in the esophagus (Fig. S3*A*), indicating a specific function of Znhit1 in lung epithelium development. The endothelial and smooth muscle cells also differentiated normally without Znhit1 (Fig. S3, *B* and *C*). These results suggest that Znhit1 in lung epithelium is dispensable for epithelial cell differentiation but exerts a regulatory function in lung branching.

### Branching failure caused by Znhit1 deletion leads to decreased proliferation and increased apoptosis of lung epithelium

**Figure 3:**
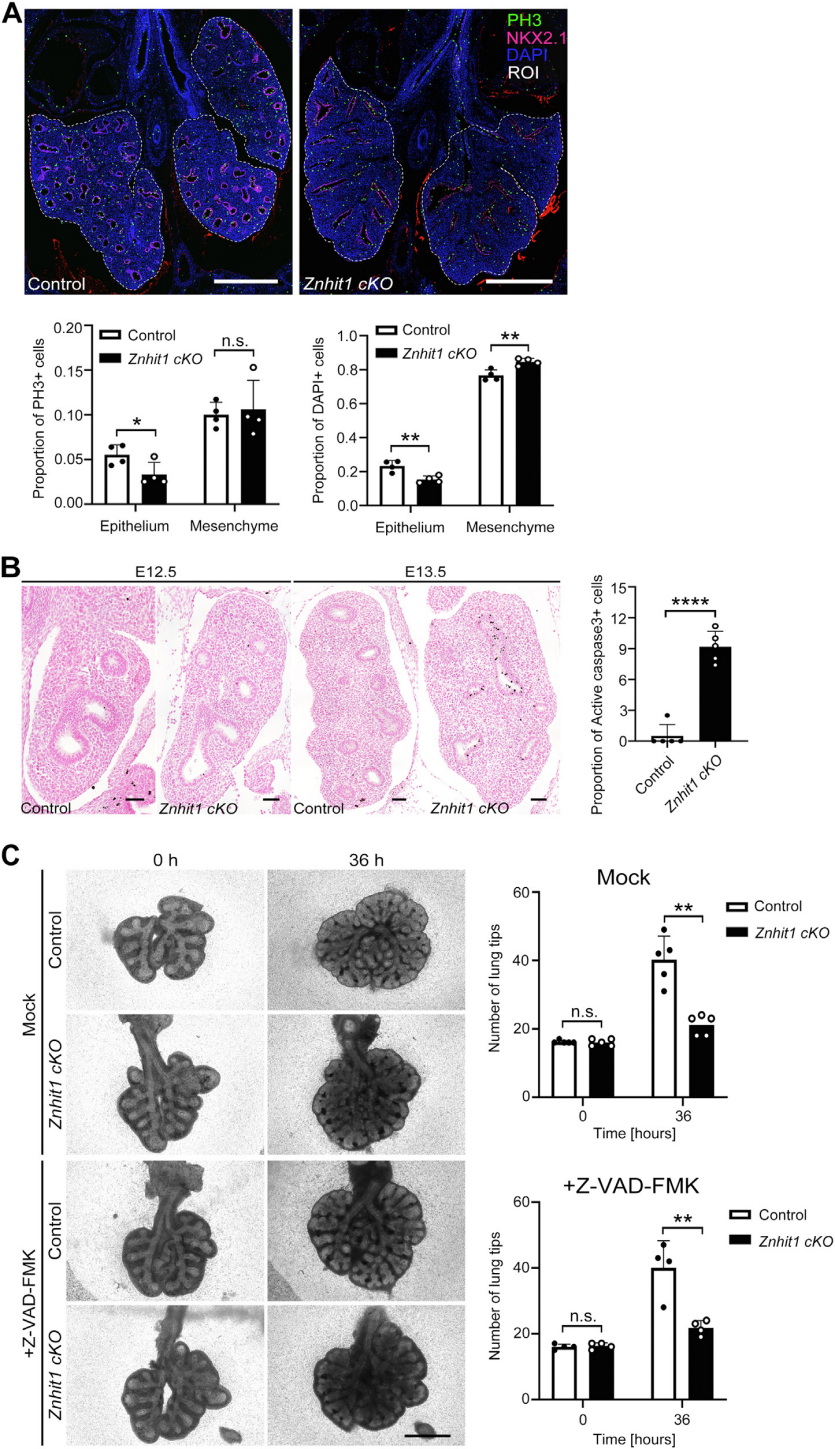
**Branching failure caused by *Znhit1* deletion leads to decreased proliferation and increased apoptosis of lung epithelium.***A*, region of interest (ROI) of phosphorylated histone H3 (PH3) staining was used for quantification. DAPI-labeled cell number quantification of epithelium and mesenchyme revealed a decreased proportion of epithelial cells in *Znhit1* conditional KO (*cKO*) lungs at embryonic day 14.5 (E14.5). Loss of Znhit1 also impairs proliferation of epithelium into late G2 and mitosis stages. Means ± SEM; ∗*p* < 0.05, ∗∗*p* < 0.01 using unpaired two-tailed Student’s *t* test. n = 4/group. The scale bars represent 500 μm. *B*, apoptotic cell staining by cleaved caspase-3 at both E12.5 and E13.5. Apoptosis of epithelium raises at E13.5 in *Znhit1 cKO* lungs. Quantification of cleaved caspase-3-positive cells at E13.5. Means ± SEM; ∗∗∗∗*p* < 0.0001 using unpaired two-tailed Student’s *t* test. n = 5/group. The scale bars represent 50 μm. *C*, 0 h and 36 h of lung explant culture with/without pan-caspase inhibitor Z-VAD-FMK and quantification of lung tips with or without Z-VAD-FMK at 0 and 36 h culture. Inhibition of apoptosis did not rescue the branching defects in *Znhit1*-deficient lungs. Means ± SEM; ∗∗*p* < 0.01 using unpaired two-tailed Student’s *t* test. n = 5/group (without inhibitor), n = 4/group (with inhibitor). The scale bars represent 1000 μm. DAPI, 4′,6-diamidino-2-phenylindole; Znhit1, zinc finger HIT-type containing 1.

Previous studies have shown that Znhit1 plays a role in p53-mediated cell apoptosis and cell cycle arrest *in vitro* (21, 22). At the cellular level, the formation of new branches is also driven by the proliferation and cytoskeleton of lung epithelial cells. To examine whether cell proliferation is affected by the deletion of *Znhit1*, we performed immunofluorescence staining and quantification analyses. Phosphorylated histone H3 (PH3) is a marker of cell proliferation and labels cells in the G2 and M phases (23), whereas Ki67 can be found throughout the cell cycle except G0 phase (24, 25). NKX2.1 was used to label fetal lung epithelial cells. Both cell number and proliferation of fetal lung epithelial and mesenchymal cells were analyzed and counted by costaining NKX2.1 and PH3 on the E14.5 fetal lung tissue sections. We found that the proliferation of epithelial cells was reduced in *Znhit1 cKO* lungs (*p* < 0.05) with unchanged cell polarity and structure (Figs. 3*A*; S4*A*). Loss of Znhit1 seemed to cause cell cycle arrest in lung epithelium as no significant differences were found in Ki67 staining (Fig. S4, *B* and *C*).

Next, we examined whether the level of cell apoptosis was changed in *Znhit1*-deficient lungs. By cleaved caspase-3 immunostaining, significantly increased apoptosis in epithelium was observed in *Znhit1*-deficient lungs (Fig. 3*B*). To test whether increased cell apoptosis affects lung branching, we dissected E12.5 lungs and performed an explant culture with pan-caspase inhibitor Z-VAD-FMK to inhibit apoptosis (Fig. S5*A*). Quantification of lung tips showed that lung branching is reduced in the *Znhit1*-deficient group, despite inhibition of apoptosis (Figs. 3*C*; S5*B*). These results suggest that reduced proliferation and increased apoptosis of lung epithelial cells induced by *cKO* of *Znhit1* cannot explain branching defects of lung epithelium.

### Znhit1 deficiency abolishes epithelium branching through activating BMP–SMADs signaling

**Figure 4:**
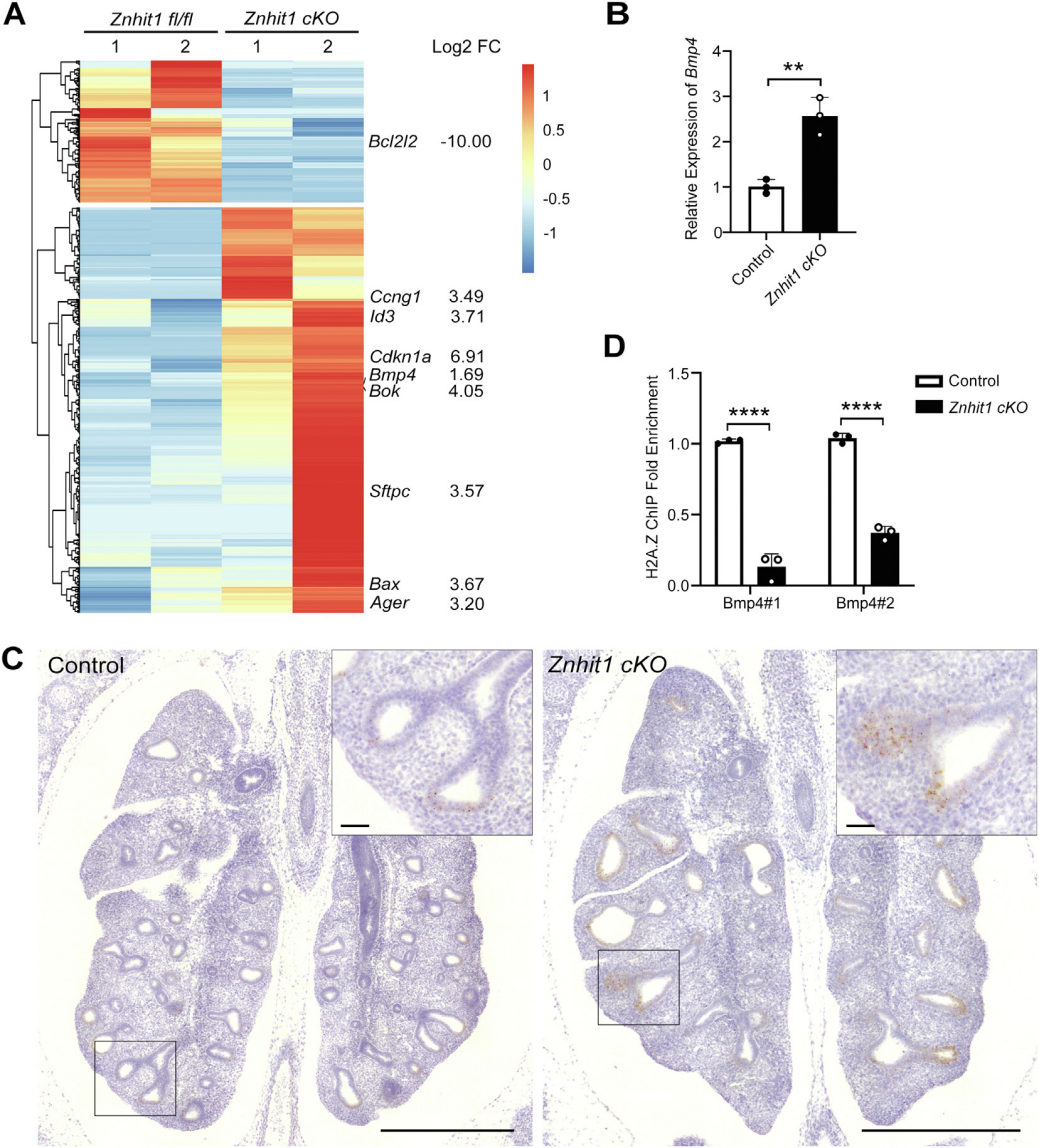
**Znhit1 deficiency abolishes epithelium branching through activating BMP–SMAD signaling.***A*, heatmap of differentially expressed genes from RNA-Seq data of EpCAM-sorted cells at embryonic day 13.5 (E13.5). *B*, quantitative RT–PCR (qRT–PCR) of *Bmp4* with RNA extracted from E13.5 control and *Znhit1*-deficient EpCAM+ sorted lung epithelial cells. Means ± SEM; ∗∗*p* < 0.01 using unpaired two-tailed Student’s *t* test. n = 3/group. *C*, RNA *in situ* of *Bmp4* in lung sections at E13.5. *Znhit1* conditional KO (*cKO*) lungs showed increased *Bmp4* expression around distal tips of epithelium. The scale bars represent 500 μm (tile scans) or 50 μm (zooms). *D*, chromatin immunoprecipitation (ChIP)–qPCR showing the fold enrichment of H2A.Z in the promoter region of *Bmp4* in control and *Znhit1 cKO* lung samples at E13.5. Means ± SEM; ∗∗∗∗*p* < 0.0001 using unpaired two-tailed Student’s *t* test. n = 3/group. BMP, bone morphogenetic protein; Znhit1, zinc finger HIT-type containing 1.

To assess the molecular consequences of Znhit1 deficiency in early lung endoderm, we isolated epithelial cells from *Znhit1*^*fl/fl*^ and *Znhit1 cKO* lungs at E13.5 to perform RNA-Seq analysis. We found that 550 genes were significantly upregulated and 192 genes were significantly downregulated by *Znhit1* deletion. Differently expressed genes were annotated to GO terms, including apoptosis, oxidative stress, and cell cycle–related clustering (Fig. S6). Consistent with increased apoptosis of lung epithelial cells in the phenotypic analysis, we found increased expression of proapoptotic Bcl family genes *BOK* and *Bax* (*p* < 0.0001) and decreased expression of *Bcl2L2* (*p* < 0.0001), which can inhibit the activity of Bax and promote cell survival (Fig. 4*A*). Moreover, *Cdkn1a* and *Ccng1*, related to cell cycle arrest, were also upregulated in the mutant epithelium, accounting for the cell proliferation disorder (Fig. 4*A*). Interestingly, *Znhit1* deletion significantly upregulated the expression of *Bmp4* in lung epithelium (Fig. 4*A*). Upregulation of *Bmp4* expression in the epithelium is further confirmed by qRT–PCR (Fig. 4*B*). As reported previously (3, 26), expression of *Bmp4* is confined to the distal tips of the airway branches as observed in control lungs by *in situ* hybridization, but the expression of *Bmp4* was elevated and pervaded throughout the epithelium of *Znhit1* mutant lungs (Fig. 4*C*). Fgf10 signaling was not significantly altered in *Znhit1* mutants (Fig. S7*A*), as were other critical genes involved in lung branching morphogenesis (Fig. S7*B*). To evaluate whether *Bmp4* is directly regulated by H2A.Z, we performed anti-H2A.Z chromatin immunoprecipitation (ChIP) assay on embryonic lung samples. H2A.Z enrichment in the promoter region of *Bmp4* significantly decreased in *Znhit1*-deficient lungs (Fig. 4*D*), supporting our hypothesis that Znhit1 promotes the incorporation of H2A.Z into the promoter region, thereby controlling *Bmp4* transcription.

### Canonical BMP–SMAD1/5/9 signaling is activated in Znhit1 *cKO* mice, and noggin treatment rescues lung budding formation in Znhit1 *cKO* lung epithelial organoids

**Figure 5:**
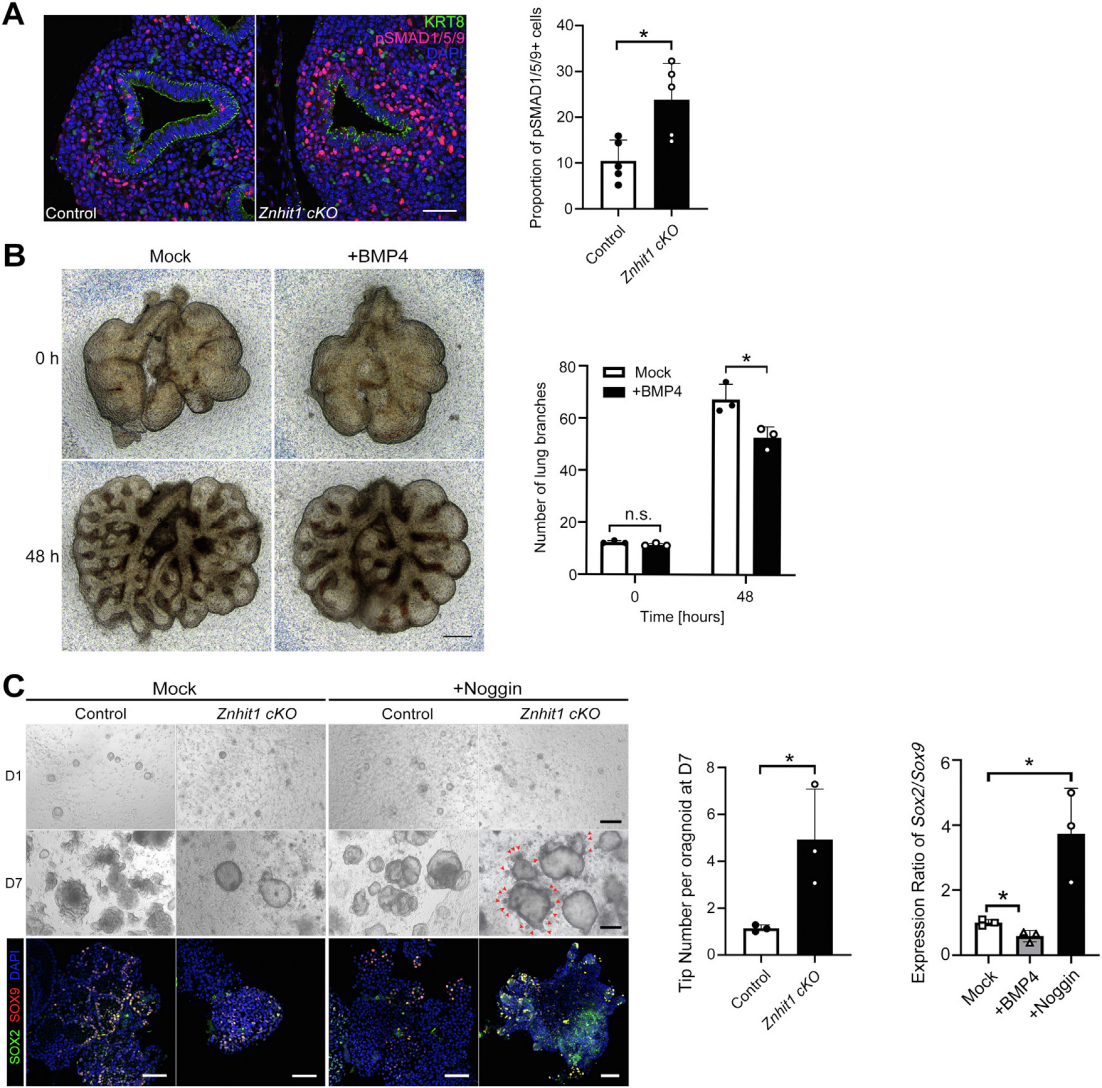
**Canonical BMP–SMAD1/5/9 signaling is activated in *Znhit1 conditional KO* (*cKO*) mice and Noggin treatment rescues lung budding formation in mutant lung epithelial organoids.***A*, phosphor-SMAD1/5/9 (pSMAD1/5/9) staining on embryonic day 13.5 (E13.5) lung sections. The scale bars represent 50 μm. Quantification of pSMAD1/5/9-positive cells. Means ± SEM; ∗*p* < 0.05 using unpaired two-tailed Student’s *t* test. n = 5/group. *B*, wildtype lungs of E12.5 were dissected and cultured *in vitro* with or without BMP4. After a culture of 48 h, lung explant with BMP4 showed expanded tips and fewer branches. The scale bars represent 200 μm. Quantification of lung branches at 0 and 48 h. Means ± SEM; ∗*p* < 0.05 using unpaired two-tailed Student’s *t* test. n = 3/group. *C*, lung organoids from E12.5 lung epithelial cells were cultured with Noggin for 7 days. Bright field scale bars represent 200 μm. Noggin treatment promoted budding formation in organoids derived from *Znhit1 cKO* epithelium. The budding number of mutant organoids was significantly increased with Noggin treatment. Means ± SEM; ∗*p* < 0.05 using unpaired two-tailed Student’s *t* test. n = 3/group. 3D lung organoids were stained SOX2 and SOX9 revealing cells at budding tips were double-positive cells. Immunofluorescence staining scale bars represent 50 μm. Quantitative RT–PCR (qRT–PCR) of *Sox2* and *Sox9* with RNA from lung epithelial organoids. Noggin treatment enhanced relative expression of *Sox2/Sox9*, whereas BMP4 treatment leads to decreased *Sox2/Sox9* expression ratio. Means ± SEM; ∗*p* < 0.05 using unpaired one-way ANOVA test. n = 3/group. BMP, bone morphogenetic protein.

To examine the activation of BMP–SMAD1/5/9 signaling in *Znhit1 cKO* lungs, we used E13.5 lung sections to stain phosphor-SMAD1/5/9 (pSMAD1/5/9), a well-established indicator of BMP signaling activity (27). Expression of pSMAD1/5/9 exhibited an expanded pattern around the dilated epithelium with much stronger fluorescence intensity than that of controls (Fig. 5*A*), which is consistent with the expanded pattern of *Bmp4*. BMP signaling is an important regulator of anterior foregut endoderm development as well as a regulator of the proximal–distal patterning of the developing lung.

Previous studies have shown that activation of BMP signaling through epigenetic changes in epithelium inhibits lung branching and proximal lung development (5, 28). Our data in lung explant culture did show a similar branching defect with BMP4 treatment (Fig. 5*B*). To determine whether the activation of BMP–SMAD1/5/9 signaling in the *Znhit1*-deficient lungs contributes to branching defects described previously, we inhibited BMP signaling by using the BMP antagonist Noggin in an *ex vivo* lung organoid culture system. Lung epithelial organoids were generated from E12.5 lung epithelial cells in a 3D culture system, and the organoids’ budding sites were identified by SOX2 and SOX9 immunofluorescent wholemount staining (Fig. 5*C*). Inhibition of BMP signaling with Noggin treatment improved budding formation of lung epithelial organoids, with loss of Znhit1 (*p* < 0.05). Consistent with SOX2+ cell loss and SOX9+ cell expansion *in vivo*, activation of BMP signaling also caused a decreased expression ratio of *Sox2/Sox9*, whereas BMP signaling inhibition increased the *Sox2/Sox9* expression ratio significantly. The organoid culture from fetal lung epithelium demonstrated that the activation of intraepithelial *Bmp4* caused by *Znhit1* ablation hindered the formation of lung buds during branching development.

## Discussion

**Figure 6:**
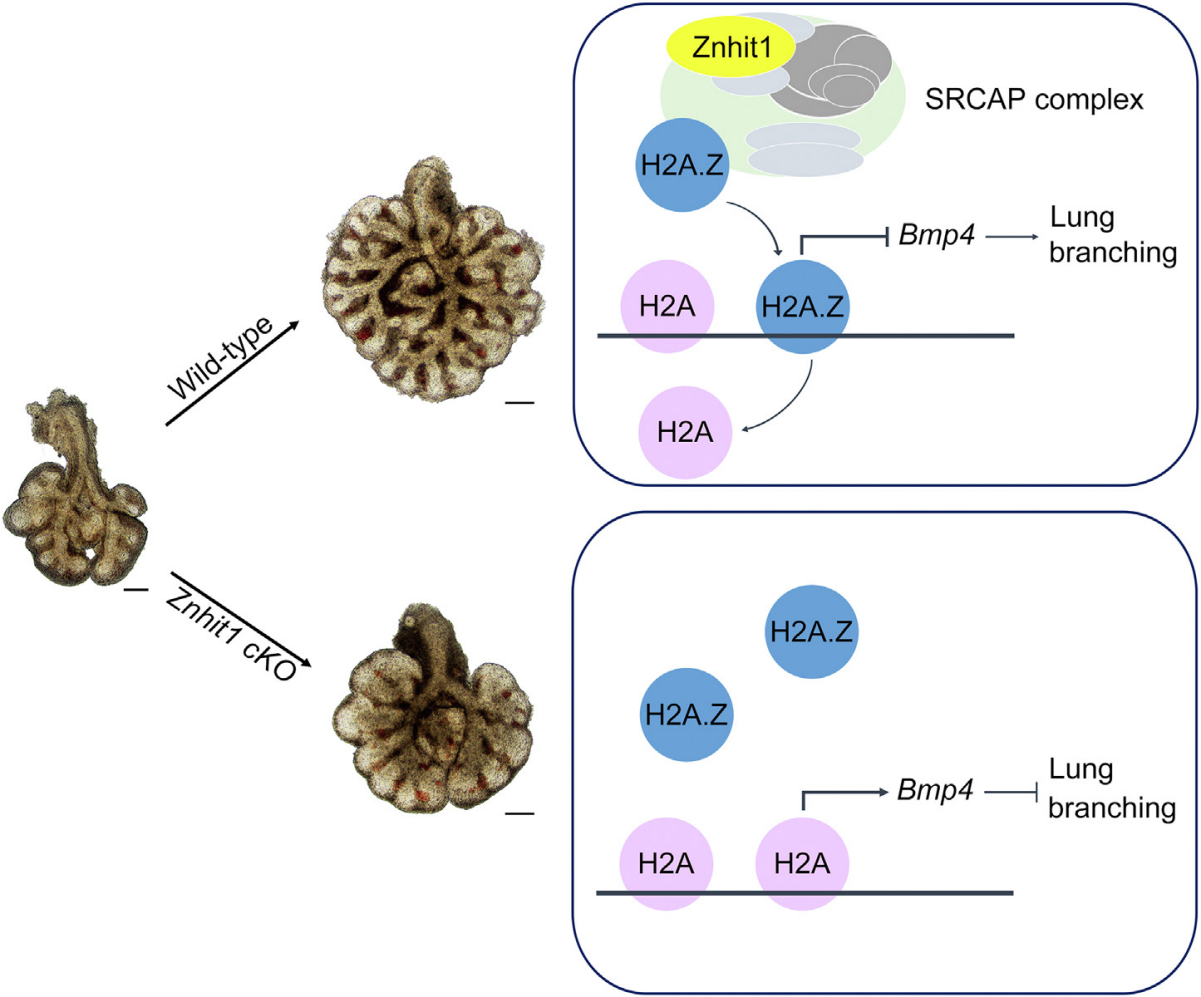
**Working model of Znhit1 regulation of embryonic lung development.** Znhit1 mediates the exchange of histone variant H2A.Z for H2A leading to repression of *Bmp4* and permitting embryonic lung branching. Loss of Znhit1 in lung epithelial increases *Bmp4* expression and causes abnormal lung branching. The scale bars represent 200 μm. Znhit1, zinc finger HIT-type containing 1.

One important issue in lung development is how epigenetic regulation is involved in cell fate determination during branching morphogenesis. Recent studies have shown that Znhit1 acts as a critical regulator of chromatin organization (12, 13, 14, 29). In this study, we have provided convincing evidence that Znhit1 is essential for lung branching morphogenesis by coordinating with the histone variant H2A.Z to regulate *Bmp4* expression (Fig. 6). Using *Znhit1 cKO* mice, we found the disrupted lung branching accompanied by decreased proliferation and increased apoptosis of lung epithelium in *Znhit1* mutants. We demonstrate that Znhit1 can repress intraepithelial *Bmp4* expression during embryonic development. Our results help to understand the mechanism by which lung development is tightly regulated under the context of chromatin.

The process of lung development, including specification, branching, and patterning, is fine tuned by key signaling pathways, such as the Wnt, BMP, and FGF pathways (30). Among these morphogen molecules, the moderate activity of BMP4 signaling plays a crucial role in regulating branching morphogenesis, and the derepression of *Bmp4* expression in lung distal epithelium would lead to lung branching defects (5). Therefore, it is important to clarify the regulatory mechanism of *Bmp4* transcription. Previous studies have shown that histone deacetylase 1/2-mediated histone deacetylation or Prmt5-mediated histone methylation is required for repressing *Bmp4* transcription (5, 28). Our work showed that excessive BMP signaling achieved by loss of Znhit1 in lung epithelium resulted in abnormal fetal lung formation, and inhibition of BMP signaling rescued budding formation in *Znhit1 cKO* lung epithelial organoids, partially. Compared with the control group, H2A.Z enrichment in the promoter region of *Bmp4* decreased dramatically in *Znhit1 cKO* embryonic lungs (Fig. 4*D*), indicating that Znhit1-dependent H2A.Z enrichment in the promoter region directly controls *Bmp4* transcription. Thus, we identify the essential role of the chromatin remodeler Znhit1 in branching morphogenesis by regulating *Bmp4* expression.

Bmp4 is not only critical for lung development but also associated with chronic obstructive pulmonary disease (COPD) (31). Inhalational exposure to tobacco smoke is one of the major risk factors for COPD (32). And previous studies reported that the induced BMP4 signaling in human airway basal cells promotes cigarette smoking–related phenotypes (31). In consideration of the crucial role of Znhit1/H2A.Z in regulating Bmp4 expression, we speculate that Znhit1/H2A.Z may also get involved in the pathogenesis of COPD.

In summary, our studies demonstrate that Znhit1 in lung epithelium represses *Bmp4* expression through mediating H2A.Z incorporation into the promoter region during branching morphogenesis. Whether the regulatory mechanism of Znhit1/H2A.Z on BMP signaling is conserved during chronic injury and the repair process of airways needs to be addressed in further studies.

## Experimental procedures

### Mice

*Znhit1*^*fl/fl*^ mice were generated by the Model Animal Research Center of Nanjing University (MARC) (12). *Shh-Cre* (The Jackson Laboratory, stock number 005622) mice were obtained from the Jackson Laboratory. For timed mating, embryonic day (E) 0.5 was determined by the presence of a copulation plug in the morning. Pregnant dams were sacrificed by CO_2_ inhalation, and embryos were harvested at different embryonic stages. All strains were maintained in C57BL/6 background. Mice were housed in pathogen-free conditions according to the protocols approved by the Institutional Animal Care and Use Committee of Fudan University.

### qRT–PCR

Total RNA was extracted with RNeasy Mini Kit (Qiagen), and the reverse transcription reaction was performed using GoScript Reverse Transcription System (Promega). qRT–PCRs were performed with GoTaq qPCR Master Mix (Promega) in triplicates on CFX96 Touch System (Bio-Rad). Primer pairs are listed in Table S1.

### Lung immunohistochemistry, immunofluorescence, and biochemistry

Embryos and embryonic lungs were fixed by 4% paraformaldehyde followed by paraffin embedding. Histological staining, immunohistochemistry, and immunofluorescence were performed on 5 μm paraffin sections. Brightfield images were obtained using a Zeiss Axio ImagerA2 microscope equipped with AxioVision Software (Carl Zeiss Microscopy, LLC). Fluorescent images were obtained using a Nikon A1Rsi inverted laser confocal microscope and Olympus FV3000 Confocal Laser Scanning Microscope. The following antibodies were used in this work: rabbit anti-ZNHIT1 (catalog no.: HPA019043; Sigma–Aldrich); mouse anti-SOX2 (catalog no.: sc-365823; Santa Cruz); rabbit anti-SOX9 (catalog no.: AB5535; Millipore); rabbit anti-CCSP (catalog no.: WRAB-3950; Seven Hills Bioreagents); guinea pig anti-proSPC (generated in the Whitsett laboratory, raised against the N terminus of proSPC); rabbit anti-HOPX (catalog no.: sc-30216; Santa Cruz); mouse anti-Alpha smooth muscle actin (catalog no.: A2547; Sigma); rabbit anti-CD31 (catalog no.: 28083-1-AP; Proteintech); rabbit anti-ZO-1 (catalog no.: 21773-1-AP; Proteintech); mouse anti-E-cadherin (catalog no.: 610182; BD Transduction); rabbit anti-PH3 (catalog no.: sc-8656-R; Santa Cruz); mouse anti-NKX2.1 (catalog no.: sc-53136; Santa Cruz); rat anti-TROMA-I (KRT8) (catalog no.: AB 531826; DSHB); mouse anti-Ki67 (catalog no.: 556003; BD Pharmingen); rabbit anti-E-cadherin (catalog no.: 3195S; Cell Signaling); rabbit anti-Active caspase-3 (catalog no.: AF835; R&D Systems); rabbit anti-pSMAD1/5/9 (catalog no.: 13820S; Cell Signaling); rabbit anti-Phospho-p44/42 MAPK (Erk1/2) (Thr202/Tyr204) (catalog no.: 4370S; Cell Signaling).

### Isolation of epithelial cells by magnetic cell sorting

E13.5 lung lobes were harvested from control and *Znhit1 cKO* embryos to isolate epithelial cells. Dispase (Corning) was used for lobe digestion. After 15 min of digestion at 37 °C, samples were transferred to 5 ml of Dulbecco's modified Eagle's medium (DMEM) containing 25 mM Hepes (Gibco) and 120 units of DNAse I (Sigma–Aldrich). Sample dissociation was performed on GentleMACS Dissociator (Miltenyi Biotec), and 40 μm nylon cell strainers (Corning) were used to get single cells. Cell suspensions that passed through the strainers were pelleted by centrifugation at 1500 rpm (433*g*) for 6 min at 4 °C and resuspended in 10 ml DMEM (with Hepes and PenStrep) and centrifuged once again. Cells were resuspended in 90 μl of autoMACS Running Buffer (Miltenyi Biotec) and 10 μl FcR Blocking Reagent and incubated at 4 °C for 10 min. Then, 10 μl of CD326 MicroBeads (Miltenyi Biotec) was added to bind epithelial cells. After incubation for 15 min at 4 °C, cells were washed twice with autoMACS Running Buffer. Cell pellets were resuspended in 500 μl of Running Buffer to pass through a 40 μm nylon filter before sorting using an AutoMACS Pro Separator (Miltenyi Biotec). CD326-positive (epithelial) and -negative (nonepithelial) cells were separated. Sorting efficiency was determined by the qRT–PCR of *Epcam* (epithelial) and *Twist2* (mesenchymal).

### RNA-Seq and analysis

RNA-Seq was performed on samples extracted from isolated lung epithelium at E13.5. The reverse transcription reaction was performed using the Ovation RNA-Seq System V2 kit (NuGEN). Sequencing and alignment were done by Berry Genomics. *Znhit1 cKO* and control samples (n = 2 each) were analyzed. The raw data were uniquely mapped to the mm10 genome by TopHat, version 1.4.1 (33). Expression values were assigned to gene level by Cufflinks, version 1.3.0 (34). All differentially expressed genes had a log2-transformed fold change >1.2 and a threshold of *p* < 0.05.

### *In situ* hybridization

Mice embryos were washed with diethyl pyrocarbonate–treated PBS and fixed in 4% paraformaldehyde overnight at 4 °C. Samples went through dehydration and paraffin embedding. *In situ* hybridization was performed on 6 μm paraffin sections with the RNA scope 2.0 kit (Advanced Cell Diagnostics). The probes used were as below: RNAscope Probe-Mm-Bmp4 (REF #401301).

### ChIP–qPCR

Samples used for ChIP were freshly harvested embryonic lungs at E13.5 (n = 3). Samples were crosslinked with 1% formaldehyde for 10 min at room temperature, then quenched with glycine, and washed with PBS. ChIP protocol was done as previously described (12). The anti-H2A.Z antibody (ab4174; Abcam) and protein A/G magnetic beads (Millipore) were used for ChIP. ChIP–qPCR was performed with two pairs of designed primers in the promoter region of *Bmp4* (Bmp4 #1 and Bmp4 #2 in Table S1).

### Lung explant culture

E11.5 lung buds were dissected from the embryos and then cultured as previously described (35) in the presence of either 250 ng/ml BMP4 (OrganRegen) or bovine serum albumin medium for 48 h.

### Lung organoid culture

E12.5 lungs were dissected from embryos and digested with prewarmed digestion buffer, containing collagenase I (Sigma) 200 U/ml and dispase II (Sigma) 6 U/ml for 15 min on a shaker at 37 °C. The cells were centrifuged at 300*g* for 4 min and washed with wash medium (DMEM supplemented with 1% penicillin/streptomycin and 1% fetal bovine serum) and cold PBS. The harvested cells were collected and embedded in Matrigel (R&D Systems) and then seeded on a 48-well plate.

Lung organoid culture medium consists of advanced DMEM/F12 (Invitrogen) (supplemented with penicillin/streptomycin, Hepes, GlutaMAX, B27, and *N*-acetylcysteine) plus 5 mM nicotinamide (Sigma), 500 ng/ml R-spondin1 (OrganRegen), 50 ng/ml epidermal growth factor(OrganRegen), 100 ng/ml FGF10 (OrganRegen), 5 μM Y27632 (Tocris), 500 nM A83-01 (Tocris), and 500 nM SB202190 (Sigma). About 100 ng/ml Noggin (OrganRegen) was added for Noggin treatment, and 200 ng/ml BMP4 was added for BMP4 treatment. Culture medium was refreshed every 3 days.

### Statistical analysis

For qRT–PCR results and cell quantification, values are expressed as the mean ± SEM. Data were analyzed using a two-tailed unpaired Student’s *t* test or one-way ANOVA test. *p* Values of 0.05 or less were considered statistically significant. Statistical analysis was performed with the GraphPad Prism 8 software (GraphPad Software, Inc).

## Data availability

RNA-Seq RAW data have been deposited to the National Center for Biotechnology Information SRA database under accession number SRP325667. All other data described in the article are contained within the main text or supporting information.

## Supporting information

This article contains supporting information.

## Conflict of interest

The authors declare that they have no conflicts of interest with the contents of this article.
