# Supplementary material for: Chromatin remodeler Znhit1 controls bone morphogenetic protein signaling in embryonic lung tissue branching
Source: J Biol Chem. 2022 Sep 14;298(10):102490. doi: 10.1016/j.jbc.2022.102490 (PMC9547297; doi:10.1016/j.jbc.2022.102490)
Supplement: Supporting Information [file mmc1.docx]

**Supporting Information**

**Chromatin Remodeler Znhit1 Controls Bone Morphogenetic Protein Signaling in Embryonic Lung Tissue Branching**

**Wei Wei^1,2^, Xiaofang Tang^1^, Ning Jiang^1^, Chao Ni^1^, Hua He^2^**, **Shenfei Sun^1^, Meng Yu^1^, Chuyue Yu^1^, Mengdi Qiu^1^, Dong Yan^1^, Zhaocai Zhou^1^, Yuanlin Song^3,4^, Hanmin Liu^2,*^, Bing Zhao^1,*^ and Xinhua Lin^1,2,4,*^**

^1^State Key Laboratory of Genetic Engineering, School of Life Sciences, Greater Bay Area Institute of Precision Medicine (Guangzhou), Zhongshan Hospital, Fudan University, Shanghai 200438, China; ^2^The Joint Laboratory for Lung Development and Related Diseases of West China Second University Hospital, Sichuan University and School of Life Sciences of Fudan University, Chengdu 610041, China; ^3^Department of Pulmonary Medicine, Zhongshan Hospital, Fudan University, Shanghai 200030, China; ^4^Shanghai Key Laboratory of Lung Inflammation and Injury, Shanghai 200032, China

**^*^**Correspondence: xlin@fudan.edu.cn (X.L.), bingzhao@fudan.edu.cn (B.Z.), or liuhm@scu.edu.cn (H.L.),

Running title: Znhit1 permits lung morphogenesis

Keywords: chromatin remodeling; lung development; branching morphogenesis; Znhit1; Bmp4

**Fig. S1 *Znhit1 cKO* embryos showed no significant differences in weight and body length.** At E16.5, the embryo weight and length were analyzed, and the weight/length ratio showed no differences between control and *Znhit1 cKO*. Means and ±SEM; using unpaired, 2-tailed Student’s t-test. *n* = 3/group.

**Fig. S2 Gross morphology of control and *Znhit1 cKO* lungs at E14.5 and E16.5.** Lung images showed that the impaired branches in *Znhit1 cKO* lungs at E14.5 evolved into cysts in the distal region at E16.5. Scale bars: 500 μm.

**Fig. S3 *Znhit1* deletion in the epithelial cells does not disrupt endothelial and smooth muscle development.**

(A) E16.5 Control and *Znhit1 cKO* esophagus sections were stained with SOX2. Scale bars: 100 μm.

(B) E18.5 Control and *Znhit1 cKO* lung sections were stained with CD31 (endothelial marker). Scale bars: 50 μm.

(C) E13.5 Control and *Znhit1 cKO* lung sections were stained with SMA (smooth muscle marker), SOX9, and KRT8 (epithelium marker). Scale bars: 200 μm.

**Fig. S4 *Znhit1* deletion in the epithelial cells does not affect cells entering the cell cycle.**

(A) E13.5 Control and *Znhit1 cKO* lung sections were stained with E-cad (E-cadherin, tight junction marker) and ZO-1 (cell apical marker). Scale bars: 50 μm.

(B) E13.5 Control and *Znhit1 cKO* lung sections were stained with Ki67 and E-cad (epithelium marker). Scale bars: 50 μm.

(C) Quantification of Ki67 positive cells at E13.5. *Znhit1 cKO* showed a normal ratio of cells in all stages of the cell cycle. Means and ±SEM; using unpaired, 2-tailed Student’s t-test. *n* = 5/group.

**Fig. S5 Inhibition of apoptosis does not rescue the branching defects of *Znhit1 cKO*.**

(A) Explant culture of lungs was embedded with paraffin and stained with cleaved caspase 3. Scale bars: 100 μm.

(B) Whole-mount staining with E-cadherin labeled the epithelium branches in lung explants cultured with apoptosis inhibitor. Scale bars: 500 μm.

**Fig. S6 Analysis of RNA sequencing data from E13.5 Control and *Znhit1 cKO* EpCAM+ lung cells.** GO enrichment barplot of RNA sequencing data from E13.5 Control and *Znhit1 cKO* lung epithelial cells.

**Fig. S7 Comparison of the expression of genes related to lung branching between Control and *Znhit1 cKO* lung samples.**

(A) pERK staining in E13.5 Control and *Znhit1 cKO* lung sections (red). Scale bars: 50 μm.

(B) qRT-PCR of key genes involved in lung branching morphogenesis. cDNA synthesized from Control and *Znhit1 cKO* whole lung RNA at E13.5 was used as the template. Means and ±SEM; ***P*<0.01 using unpaired, 2-tailed Student’s t-test. Control: *n* = 2; *Znhit1 cKO*: *n* = 3.

**Table S1 Primers list**

| **Primers for ChIP-qPCR** | | |
| --- | --- | --- |
|  | **Forward** | **Reverse** |
| ***Bmp4#1*** | AGGCCACCCTTTAAACCAAT | AATCACCATTTACCCCGAGT |
| ***Bmp4#2*** | GCAGGCTCCAAGACGGTTTA | AGCTAGGTGAGTTCGGCATC |
| **Primers for qPCR** | | |
| ***Gapdh*** | GCCCTTCCACAATGCCAAAG | ATCACCATCTTCCAGGAGCGAG |
| ***Znhit1*** | CAGACGGCGAGACAAGTTC | TGGGCAAGAGGCTACCTCA |
| ***Bmp4*** | TTCCTGGTAACCGAATGCTGA | CCTGAATCTCGGCGACTTTTT |
| ***Sox2*** | AACGGCAGCTACAGCATGATGC | CGAGCTGGTCATGGAGTTGTAC |
| ***Sox9*** | CACACGTCAAGCGACCCATGAA | TCTTCTCGCTCTCGTTCAGCAG |
| ***Shh*** | AAAGCTGACCCCTTTAGCCTA | TTCGGAGTTTCTTGTGATCTTCC |
| ***Spry2*** | TCCAAGAGATGCCCTTACCCA | GCAGACCGTGGAGTCTTTCA |
| ***Axin2*** | TGACTCTCCTTCCAGATCCCA | TGCCCACACTAGGCTGACA |
| ***Gli1*** | CCAAGCCAACTTTATGTCAGGG | AGCCCGCTTCTTTGTTAATTTGA |
